# Prognostic value of cross‐lineage expression of the myeloid‐associated antigens CD13 and CD33 in adult B‐lymphoblastic leukemia: A large real‐world study of 1005 patients

**DOI:** 10.1002/cam4.5739

**Published:** 2023-03-23

**Authors:** Hongyan Liao, Hongli Lai, Jiao Chen, Xiao Shuai, Xin Zhang, Ying Yang, Mengyuan Lyu, Qin Zheng

**Affiliations:** ^1^ Department of Laboratory Medicine West China Hospital of Sichuan University Chengdu China; ^2^ Department of Hematology West China Hospital of Sichuan University Chengdu China

**Keywords:** B‐ALL, BCR::ABL1, CD13, CD33, cross‐lineage expression, MRD, prognosis

## Abstract

**Background:**

Cross‐lineage expression of the myeloid‐associated antigens CD13/CD33 is common in adult B‐lymphoblastic leukemia (B‐ALL) patients, yet its prognostic value is still controversial.

**Methods:**

We conducted a retrospective study of 1005 de novo adult B‐ALL patients from January 2009 to December 2019 in our hospital. Logistic and Cox regression were used to analyze the prognostic value of CD13/CD33 expression in B‐ALL. A Cox regression model was established to predict overall survival (OS) for B‐ALL patients.

**Results:**

Of the 1005 B‐ALL patients, 53.7% (*n* = 540) aberrantly expressed CD13/CD33 (CD13/CD33^+^). Patients in the CD13/CD33^+^ group showed a higher incidence of BCR::ABL1 rearrangement and minimal/measurable residual disease (MRD) positivity but similar complete remission rate, relapse‐free survival, mortality, and OS with CD13/CD33^‐^. CD13/CD33^+^ patients had a higher risk of MRD positivity than CD13/CD33^‐^ patients. Notably, CD13/CD33^+^ patients who underwent tyrosine kinase inhibitor (TKI) therapy had a better long‐term prognosis than those without TKI experience. Sex, group based on CD13/CD33 expression and TKI experience and white blood cell count were variables independently associated with OS. The Cox regression model integrating these three variables showed a moderate performance for OS prediction (C‐index: 0.724).

**Conclusions:**

In real‐world practice, CD13/CD33 expression can predict the risk of MRD in patients without TKI experience, but has no adverse effect on the prognosis of adult B‐ALL patients. Incorporating CD13/CD33 into the standard antibody panels of B‐ALL diagnosis and MRD measurements can help predict relapse risk and decisions on therapy options.

## 1 INTRODUCTION

Acute lymphoblastic leukemia (ALL) is a hematological malignancy characterized by the clonal proliferation of lymphoid progenitor cells.^1^ B‐lymphoblastic leukemia (B‐ALL) is the primary type of ALL, accounting for 85%–90% of pediatric ALL cases and 75% of adult ALL cases.^2^ B‐ALL has a good prognosis in children but a less favorable prognosis in adults. Infancy, older patient age, higher white blood cell (WBC) count, presence of central nervous system (CNS) disease at diagnosis, slow response to initial therapy as assessed by morphological examination of peripheral blood (PB), and/or bone marrow (BM), and the presence of minimal/measurable residual disease (MRD) after therapy are all associated with adverse prognosis of B‐ALL.^1^, ^3^, ^4^ In addition, molecular characteristics such as BCR::ABL1 and TCF3‐HLF fusion genes were defined as high‐risk factors.^1^ Despite the many recognized prognostic factors and the identification of different prognostic subgroups, the outcome of B‐ALL patients still shows considerable heterogeneity. Among B‐ALL patients in the standard‐risk group, 20% experience relapse or drug resistance, leading to rapid disease progression and even death.^5^, ^6^, ^7^ Therefore, identifying more alternative laboratory indicators to assess disease prognosis is of invaluable clinical significance.

The diagnosis and risk stratification of B‐ALL require the integration of clinical and laboratory findings, including cytomorphology, immunophenotype, molecular characteristics, and cytogenetics. Immunophenotyping via flow cytometry (FCM) allows distinguishing the lineage and developmental stage of leukemic cells and thus plays a key role in subtype classification, efficacy assessment, and outcome prediction.^8^, ^9^, ^10^, ^11^ Leukemic cells always have aberrant immunophenotypes. For example, myeloid‐associated antigens (MyAgs) can be coexpressed in 20% ~ 40% of ALL patients.^12^, ^13^ However, the prognostic value of CD13/CD33 in B‐ALL patients is still controversial. Earlier studies suggested an inferior outcome for MyAg+ ALL patients,^14^, ^15^, ^16^, ^17^ while other reports failed to confirm such prognostic correlation based on high‐dose chemotherapies.^18^, ^19^, ^20^, ^21^ This discrepancy is possibly due to the small numbers of cases, variations in the regimens, limited follow‐up time, interaction with cytogenetic abnormalities, and differences in the definition of MyAg positivity.^18^, ^22^ Therefore, larger and more comprehensive studies in the real world addressing the aberrant expression of CD13/CD33 in adult B‐ALL patients are needed.

Herein, we explored the influence of CD13/CD33 on the prognosis of adult B‐ALL based on a large single‐center retrospective analysis of 1005 real‐world patients during a 10‐year period. Notably, we established a predictive model for B‐ALL prognosis combining the laboratory and clinical characteristics with treatment regimens and patient outcomes.

## 2 MATERIALS AND METHODS

### 2.1 Patient recruitment

A total of 1005 de novo B‐ALL patients before treatment were consecutively recruited from West China Hospital of Sichuan University from January 2009 to December 2019. All participants had an unequivocal diagnosis of B‐ALL based on morphology, immunology, cytogenetics, and molecular biology analysis according to the 2008 or 2016 World Health Organization classification criteria,^9^, ^23^ no other malignant tumors, age equal to or older than 14 years and complete clinical data. All samples were analyzed at initial presentation at our center.

### 2.2 Ethics statement

Protocols were conducted in accordance with the Declaration of Helsinki. The study protocol was approved by the Ethics Committee of West China Hospital of Sichuan University. All enrolled patients provided signed written informed consent.

### 2.3 Immunophenotypic analysis by FCM

Fresh heparinized PB and/or BM aspirates were processed for FCM immunophenotyping following the lyse/wash surface or surface/intracellular staining procedure. Antibodies against the following antigens were used specifically to profile the B‐precursor cells: CD10, CD19, CD20, CD22, cCD79a, CD34, CD38, HLA‐DR, and CD45 (Becton Dickinson). Additional antibodies were used to characterize the leukemia cells, including T lymphocyte lineage‐associated antigens cytoplasmic CD3 (cCD3), CD3 and CD5 and myeloid lineage‐associated antigens CD13, CD33, CD117, and myeloperoxidase (Becton Dickinson). One hundred thousand events were acquired from each tube by a 3‐laser/10‐color FACSCanto II cytometer (BD Biosciences). The instrument setup and compensation matrix were established using CS&T beads (BD Biosciences) per the manufacturer's recommendations.

Data analysis was performed using FACSDiva™ software (BD Biosciences). Antigen expression was considered positive when the proportion of leukemia cells with the corresponding surface marker was more than 20% and the cytoplasmic/nuclear marker was more than 10%. All data were analyzed in dot plots. Dead cells and debris were excluded by forward scatter (FSC)/side scatter (SSC). Doublets were excluded on FSC‐A/FSC‐H dot plots. All B‐lineage cells were roughly gated by CD45/SSC or CD19/SSC. B‐ALL was divided into three subtypes, pro‐B, pre‐B, and common‐B, representing the sequential degree of precursor B cell differentiation. MRD‐positive events were identified by homogeneous coexistence of multiple aberrant antigen expression in an accumulation of at least 10‐event clusters different from normal hematogones and showing properties of a leukemia‐associated immunophenotype. MRD was calculated as the percentage of MRD‐positive events in the total number of viable nucleated cells. The cut‐off value for MRD positivity was 0.01%.

### 2.4 Molecular genetic analysis by quantitative real‐time PCR (RT–PCR)

RNA was prepared using a QIAamp RNA Blood Mini Kit (QIAGEN) according to the manufacturer's protocol. All fusion genes (BCR::ABL1, E2A‐PBX1, SIL‐TAL1, TEL‐AML1, MLL‐AF4, CALM‐AF10, HOX11, E2A‐HLF, etc.) were detected by RT–PCR using a Leukemia Related Fusion Gene Detection Kit (FQ‐RT–PCR) (Yqbiomed). PCR was performed by a Roche LightCycler 480 Real‐Time PCR System (Roche Diagnostics) or QuantStudio 5 (Applied Biosystems). The thermocycling conditions consisted of predenaturation at 42°C for 5 min and 94°C for 3 min, followed by 40 cycles of 94°C for 15 s and 60°C for 60 s. The quantity of the BCR::ABL1 transcript was calculated as a ratio of BCR::ABL1 copy number relative to ABL copies.

### 2.5 Development of the predictive model

Cox regression was used to build a model predicting overall survival (OS). Univariate and multivariate Cox regression analyses were performed for preliminary variable selection. On the basis of the candidate variables, Cox models with all possible combinations of these variables were constructed using the “survival” package in R software. The concordance index (C‐index) and calibration curves were used to evaluate the overall performance of the models. The optimal model was based on the consideration of both the number of included variables and the C‐index and then illustrated in the form of a nomogram.

### 2.6 Definitions

Complete remission (CR) was defined by <5% leukemia cells in a regenerated BM aspirate, lack of extramedullary leukemia and PB platelet and neutrophil counts of >100 × 10^9^/L and 1.5 × 10^9^/L, respectively, according to the National Comprehensive Cancer Network (NCCN) guidelines, version 4.2021. OS was defined as the survival period from initial diagnosis to death or survival at the last follow‐up (censored). Relapse was defined as ≥5% leukemia cells in the BM or PB after CR. Relapse‐free survival (RFS) was defined as the survival period until recurrence.

### 2.7 Statistical analysis

Categorical variables and continuous variables were compared by the chi‐square test or Fisher's exact test and Student's *t‐* test, respectively. For categorical outcomes, univariate and multivariate logistic regression analyses were carried out. Odds ratios (ORs) with corresponding 95% confidence intervals (CIs) were computed. For time‐dependent outcomes, univariate and multivariate Cox regression analyses were conducted. The hazard ratio (HR) with the corresponding 95% CI was calculated. Estimates of median RFS and OS were calculated with the use of the reverse the Kaplan–Meier method. A log‐rank test was performed to compare RFS and OS. Two‐sided *p* values <0.05 were considered significantly different. SPSS software (IBM) was used for all analyses.

## 3 RESULTS

### 3.1 Basic characteristics of enrolled patients

A total of 1005 patients were finally included in this work. There were 501 males and 504 females, with a median age of 35 years (range, 22–46 years) and a median WBC count of 11.79 × 10^9^/L (range, 3.35–54.14 × 10^9^/L). Anemia (Hb < 100 g/L for males and < 90 g/L for females or adolescents) and thrombocytopenia (platelet < 100 × 10^9^/L) were observed in 54.3% and 63.3% of the patients, respectively. A total of 9.1% (*n* = 91) had CNS involvement at initial diagnosis or during the following treatment. Of all enrolled B‐ALL patients, common‐B accounting for 58.5% was the predominant subtype, followed by pro‐B (34.5%) and pre‐B (7.0%).

Aberrant CD13 and CD33 expression was observed in 49.0% and 22.6% of the entire study population, respectively. As the coexpression of CD13 and CD33 showed no difference in the prognostic implication compared with individual positivity for CD13 or CD33 (data not shown), the following analysis was reported according to the presence of CD13 and/or CD33. In total, 53.7% (*n* = 540) of the study subjects had CD13 and/or CD33 expression, according to which they were divided into the CD13/CD33^+^ and CD13/CD33^−^ groups. The CD13/CD33^+^ group showed differential B‐ALL subtypes with a higher common‐B proportion (*p* = 0.001), lower level of lactate dehydrogenase (LDH) (*p* = 0.030) and higher positivity ratio of CD34 (*p* < 0.001). In 702 cases with available molecular analysis results, 45.6% of the total were positive for the BCR::ABL1 rearrangement. Interestingly, in CD13/CD33^+^ patients, the incidence of BCR::ABL1 was higher (54.5%). Alternatively, CD13 and/or CD33 positivity was significantly correlated with the presence of BCR::ABL1 (*p* < 0.001), although no difference was detected as to the transcript forms (p190 and p210) between the two groups. The proportions of other cytogenetics (MLL‐AF4, TEL‐AML1, E2A‐PBX1, etc.) (*p* = 0.045) were also distinct between the two groups, with a higher positive ratio of TEL‐AML1 and a lower positive ratio for MLL‐AF4 and E2A‐PBX1 in the CD13/CD33^+^ group. The basic characteristics of the enrolled patients are shown in Table S1.

### 3.2 Univariate regression analysis of the effect of CD13/CD33 expression on patient outcomes

To determine the effect of CD13/CD33 expression on real‐world B‐ALL patients, we compared the parameters reflecting clinical courses and outcomes between the CD13/CD33^+^ and CD13/CD33^−^ groups (Table 1). Notably, patients in the CD13/CD33^+^ group were more likely to have MRD (OR = 1.45, 95% CI: 1.006–2.091, *p* = 0.046). The two groups showed no significant differences in other outcome measures, including CR1, RFS, and OS.

**TABLE 1:** The effect of CD13/CD33 expression on outcome measures of adult B‐ALL patients.

Outcome measures	All included patients (*n* = 1005)				Univariate regression analysis	
	Overall	CD13/CD33^+^ (*n* = 540)	CD13/CD33^−^ (*n* = 465)	*p* Value^a^	OR/HR (95% CI)	*p* Value^b^
CR1, *n* (%)	362 (76.9)	194 (78.2)	168 (75.3)	0.458	1.176 (0.766–1.805)	0.458
MRD, *n* (%)	203 (42.6)	118 (46.8)	85 (37.8)	**0.046**	1.450 (1.006–2.091)	**0.046**
CNS involvement, *n*(%)	91 (21.2)	45 (19.7)	46 (22.8)	0.442	0.834 (0.525–1.325)	0.442
Time to CNS involvement, months	5.00 (2.00–12.00)	6.00 (2.00–12.00)	5.00 (2.00–11.50)	0.580	0.855 (0.560–1.305)	0.468
Relapse, *n* (%)	224 (54.4)	115 (53.5)	109 (55.3)	0.708	0.928 (0.630–1.369)	0.708
RFS, months	7.00 (3.00–19.00)	7.00 (3.00–19.00)	6.00 (3.00–21.00)	0.973	0.988 (0.742–1.316)	0.935
Death, *n* (%)	77 (12.4)	35 (10.4)	42 (14.7)	0.107	0.675 (0.418–1.090)	0.108
OS, months	9.00 (4.0–19.00)	10.00 (4.00–20.00)	9.00 (3.00–19.00)	0.074	0.654 (0.408–1.048)	0.077

*Note*: Categorical variables are presented as numbers (percentages). Continuous variables without normal distributions were expressed as the median with the 25th and 75th percentiles. Bold values indicate significant differences were identified.

Abbreviations: CR1, complete remission after first induction; CNS, central nervous system; MRD, minimal residual disease; OS, overall survival; RFS, relapse‐free survival.

^a^ *p* Value of the difference analysis between two groups by chi‐square test, Fisher's exact test or Student's *t‐*test.

^b^ *p* Value of the univariate regression analysis for identifying the predictive value of CD13/CD33 on B‐ALL patient outcomes.

### 3.3 Univariate analysis of the prognostic value of the group of B‐ALL patients

The finding that CD13 and/or CD33 positivity was correlated with BCR::ABL1, a well‐recognized treatment target in the tyrosine kinase inhibitor (TKI) regimen, prompted us to examine its prognostic value in B‐ALL in depth. Thus, we further divided the patients into four subgroups, CD13/CD33^−^‐TKI, CD13/CD33^−^‐non‐TKI, CD13/CD33^+^‐TKI, and CD13/CD33^+^‐non‐TKI, based on CD13/CD33 expression and TKI experience. A univariate analysis was performed to explore the prognostic value of group (Table 2).

**TABLE 2:** Univariate regression analysis of the prognostic value of the B‐ALL patient group.

	Outcome measures	Variable	OR/HR (95% CI)	*p* Value
Univariate logistic regression	CR1	Group^a^	/	**0.001**
		CD13/CD33^−^‐TKI vs. CD13/CD33^−^‐non‐TKI	2.056 (0.992–4.259)	0.053
		CD13/CD33^+^‐non‐TKI vs. CD13/CD33^−^‐non‐TKI	0.789 (0.454–1.370)	0.400
		CD13/CD33^+^‐TKI vs. CD13/CD33^−^‐non‐TKI	**2.941 (1.467–5.894)**	**0.002**
		CD13/CD33^−^‐TKI vs. CD13/CD33^+^‐TKI	0.699 (0.299–1.634)	0.408
		CD13/CD33^+^‐non‐TKI vs. CD13/CD33^+^‐TKI	**0.268 (0.133–0.542)**	**<0.001**
		CD13/CD33^−^‐TKI vs. CD13/CD33^+^‐non‐TKI	**2.604 (1.248–5.435)**	**0.011**
	MRD	Group	/	0.22
		CD13/CD33^−^‐TKI vs. CD13/CD33^−^‐non‐TKI	1.313 (0.732–2.355)	0.36
		CD13/CD33^+^‐non‐TKI vs. CD13/CD33^−^‐non‐TKI	**1.720 (1.001–2.956)**	**0.049**
		CD13/CD33^+^‐TKI vs. CD13/CD33^−^‐non‐TKI	1.531 (0.907–2.583)	0.111
		CD13/CD33^−^‐TKI vs. CD13/CD33^+^‐TKI	0.858 (0.480–1.532)	0.604
		CD13/CD33^+^‐non‐TKI vs. CD13/CD33^+^‐TKI	1.124 (0.657–1.923)	0.670
		CD13/CD33^−^‐TKI vs. CD13/CD33^+^‐non‐TKI	0.763 (0.420–1.386)	0.375
	Relapse rate	Group	/	0.119
		CD13/CD33^−^‐TKI vs. CD13/CD33^−^‐non‐TKI	0.758 (0.411–1.395)	0.373
		CD13/CD33^+^‐non‐TKI vs. CD13/CD33^−^‐non‐TKI	1.147 (0.643–2.047)	0.642
		CD13/CD33^+^‐TKI vs. CD13/CD33^−^‐non‐TKI	0.595 (0.343–1.032)	0.065
		CD13/CD33^−^‐TKI vs. CD13/CD33^+^‐TKI	1.273 (0.686–2.363)	0.445
		CD13/CD33^+^‐non‐TKI vs. CD13/CD33^+^‐TKI	**1.927 (1.071–3.469)**	**0.029**
		CD13/CD33^−^‐TKI vs. CD13/CD33^+^‐non‐TKI	0.661 (0.347–1.258)	0.207
	Mortality	Group	/	0.101
		CD13/CD33^−^‐TKI vs. CD13/CD33^−^‐non‐TKI	0.739 (0.355–1.537)	0.418
		CD13/CD33^+^‐non‐TKI vs. CD13/CD33^−^‐non‐TKI	0.872 (0.473–1.607)	0.661
		CD13/CD33^+^‐TKI vs. CD13/CD33^−^‐non‐TKI	**0.387 (0.180–0.829)**	**0.015**
		CD13/CD33^−^‐TKI vs. CD13/CD33^+^‐TKI	1.911 (0.787–4.638)	0.153
		CD13/CD33^+^‐non‐TKI vs. CD13/CD33^+^‐TKI	**2.256 (1.025–4.967)**	**0.043**
		CD13/CD33^−^‐TKI vs. CD13/CD33^+^‐non‐TKI	0.847 (0.396–1.812)	0.668
Univariate Cox regression	OS	Group	/	0.058
		CD13/CD33^−^‐TKI vs. CD13/CD33^−^‐non‐TKI	0.683 (0.344–1.354)	0.274
		CD13/CD33^+^‐non‐TKI vs. CD13/CD33^−^‐non‐TKI	0.825 (0.464–1.467)	0.513
		CD13/CD33^+^‐TKI vs. CD13/CD33^−^‐non‐TKI	**0.353 (0.166–0.755)**	**0.007**
		CD13/CD33^−^‐TKI vs. CD13/CD33^+^‐TKI	1.931 (0.813–4.586)	0.136
		CD13/CD33^+^‐non‐TKI vs. CD13/CD33^+^‐TKI	**2.355 (1.069–5.098)**	**0.033**
		CD13/CD33^−^‐TKI vs. CD13/CD33^+^‐non‐TKI	0.827 (0.407–1.681)	0.600

Abbreviations: CR1, complete remission after first induction; CI, confidence interval; HR, hazard ratio; MRD, minimal residual disease; OS, overall survival; OR, odds ratio. Bold values indicate significant differences were identified.

^a^ According to CD13/CD33 expression and whether patients were undergoing TKI therapy, patients were categorized into four groups: CD13/CD33 negative without TKI treatment group (CD13/CD33^−^‐non‐TKI), CD13/CD33 negative with TKI treatment group (CD13/CD33^−^‐TKI), CD13/CD33 positive without TKI therapy group (CD13/CD33^+^‐non‐TKI), and CD13/CD33 positive with TKI treatment group (CD13/CD33^+^‐TKI).

The CD13/CD33^+^‐non‐TKI subgroup showed a higher risk of relapse than the CD13/CD33^+^‐TKI subgroup (OR = 1.927, 95% CI: 1.071–3.469, *p* = 0.029). Interestingly, the CD13/CD33^+^‐non‐TKI subgroup showed a higher risk of MRD positivity than CD13/CD33^−^‐non‐TKI (OR = 1.720, 95% CI: 1.001–2.956, *p* = 0.049), while there was no significant difference observed in comparisons of CD13/CD33^−^‐TKI and CD13/CD33^+^‐TKI. Meanwhile, the CD13/CD33^+^‐TKI subgroup showed lower mortality than patients without TKI experience regardless of CD13/CD33 expression. The incidence of CNS involvement, time to involvement, and RFS did not reach statistical significance in the pairwise comparisons among all subgroups via univariate analysis.

Of the total 320 BCR::ABL1^+^ patients, 205 had TKI treatment experience, 31 were never treated with TKI and the remaining 84 had no treatment information available. To eliminate the effect of the inconsistency between BCR::ABL1 and TKI experience, we also divided the patients into four subgroups according to the CD13/CD33 expression and BCR::ABL1 fusion gene and performed a univariate regression analysis (Table S3). Univariate regression analysis revealed that this variable was not significantly associated with all the outcome measures (*p* > 0.10), except for relapse rate (*p* = 0.076).

**FIGURE 1:**
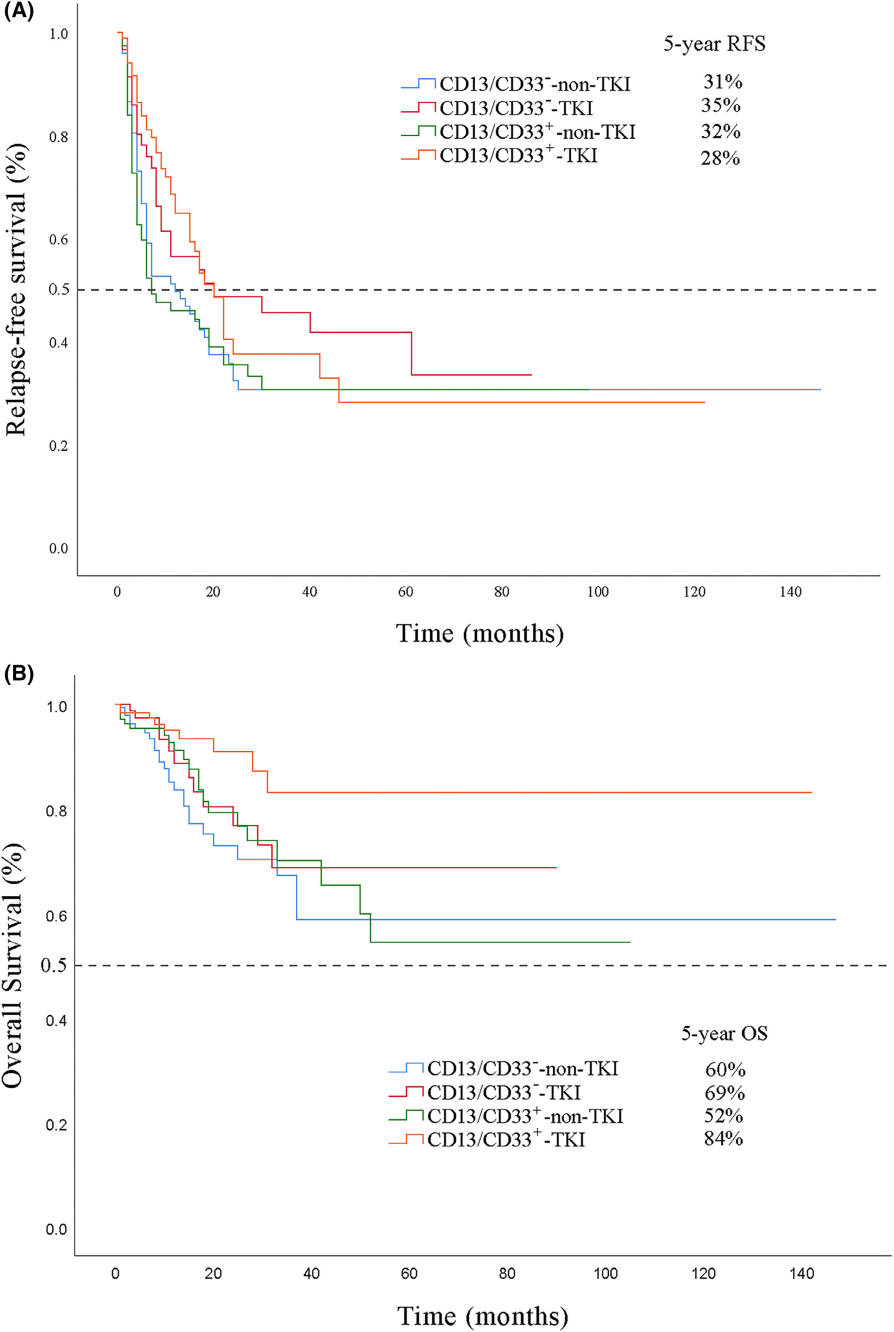
Survival outcomes for patients with B‐cell acute lymphoblastic leukemia. Kaplan–Meier curves for (A) relapse‐free survival and (B) overall survival of the study population.

In Kaplan–Meier analyses, the 5‐year RFS rates of the CD13/CD33^−^‐TKI, CD13/CD33^−^‐non‐TKI, CD13/CD33^+^‐TKI, and CD13/CD33^+^‐non‐TKI subgroups were 35%, 31%, 28%, and 32%, respectively; the 5‐year OS rates of these subgroups were 69%, 60%, 84%, and 52%. Although the CD13/CD33^+^‐TKI subgroup seemed to show a better OS than the CD13/CD33^−^‐non‐TKI subgroup (*p* = 0.028), the RFS between these two groups were not statistically different (*p* = 0.190). Furthermore, no significant differences were observed in the pairwise comparisons of RFS and OS among all other subgroups (Figure 1A,B).

### 3.4 Multivariate analysis of short‐term and long‐term prognosis for B‐ALL patients

Multivariate analyses were conducted to explore the potential independent prognostic factors of B‐ALL patients (Table 3).

**TABLE 3:** Multivariate logistic regression and Cox regression analysis of short‐term and long‐term prognosis for adult B‐ALL patients.

	Outcome measures	Variable	OR/HR (95% CI)	*p* Value
Multivariate logistic regression	CR1	Group^a^		**<0.001**
		CD13/CD33^−^‐TKI vs. CD13/CD33^−^‐non‐TKI	**2.687 (1.234–5.851)**	**0.013**
		CD13/CD33^+^‐non‐TKI vs. CD13/CD33^−^‐non‐TKI	0.704 (0.399–1.241)	0.225
		CD13/CD33^+^‐TKI vs. CD13/CD33^−^‐non‐TKI	**3.043 (1.503–6.163)**	**0.002**
		CD13/CD33^−^‐TKI vs. CD13/CD33^+^‐TKI	0.883 (0.365–2.139)	0.783
		CD13/CD33^+^‐non‐TKI vs. CD13/CD33^+^‐TKI	**0.231 (0.112–0.478)**	**<0.001**
		CD13/CD33^−^‐TKI vs. CD13/CD33^+^‐non‐TKI	**3.820 (1.705–8.559)**	**0.001**
		WBC	**0.996 (0.994–0.999)**	**0.005**
	MRD	B‐ALL subtypes		**0.029**
		common‐B vs. pro‐B	1.203 (0.804–1.798)	0.369
		pre‐B vs. pro‐B	**0.232 (0.065–0.825)**	**0.024**
		pre‐B vs. common‐B	**0.193 (0.055–0.671)**	**0.01**
		Age	**0.987 (0.975–1.000)**	**0.043**
	Mortality	Sex(Male vs. Female)	**1.783 (1.073–2.963)**	**0.026**
		CD10(+ vs. ‐)	**0.509 (0.284–0.913)**	**0.023**
		WBC	**1.003 (1.000–1.005)**	**0.031**
Multivariate Cox regression	RFS	Group		**0.021**
		CD13/CD33^−^‐TKI vs. CD13/CD33^−^‐non‐TKI	0.626 (0.389–1.006)	0.053
		CD13/CD33^+^‐non‐TKI vs. CD13/CD33^−^‐non‐TKI	1.081 (0.724–1.612)	0.704
		CD13/CD33^−^‐non‐TKI vs. CD13/CD33^+^‐TKI	**1.631 (1.065–2.500)**	**0.025**
		CD13/CD33^−^‐TKI vs. CD13/CD33^+^‐TKI	1.021 (0.619–1.685)	0.935
		CD13/CD33^+^‐non‐TKI vs. CD13/CD33^+^‐TKI	**1.754 (1.130–2.754)**	**0.013**
		CD13/CD33^−^‐TKI vs. CD13/CD33^+^‐non‐TKI	**0.579 (0.355–0.945)**	**0.029**
		WBC	**1.003 (1.002–1.005)**	**<0.001**
	OS	Group		**0.048**
		CD13/CD33^−^‐TKI vs. CD13/CD33^−^‐non‐TKI	0.675 (0.334–1.364)	0.274
		CD13/CD33^+^‐non‐TKI vs. CD13/CD33^−^‐non‐TKI	0.948 (0.513–1.754)	0.866
		CD13/CD33^+^‐TKI vs. CD13/CD33^−^‐non‐TKI	**0.354 (0.164–0.764)**	**0.008**
		CD13/CD33^−^‐TKI vs. CD13/CD33^+^‐TKI	1.905 (0.800–4.538)	0.145
		CD13/CD33^+^‐non‐TKI vs. CD13/CD33^+^‐TKI	**2.676 (1.195–5.995)**	**0.017**
		CD13/CD33^−^‐TKI vs. CD13/CD33^+^‐non‐TKI	0.712 (0.337–1.504)	0.373
		Sex(Male vs. Female)	**2.127 (1.263–3.585)**	**0.005**
		WBC	**1.004 (1.002–1.006)**	**<0.001**

Abbreviations: CR1, complete remission after first induction; CI, confidence interval; Hb, hemoglobin; HR, hazard ratio; MRD, minimal residual disease; OR, odds ratio; OS, overall survival; PLT, platelet; RFS, relapse‐free survival; WBC, white blood cell. Bold values indicate significant differences were identified.

^a^ According to CD13/CD33 expression and whether patients were undergoing TKI therapy, patients were categorized into four groups: CD13/CD33 negative without TKI treatment group (CD13/CD33^−^‐non‐TKI), CD13/CD33 negative with TKI treatment group (CD13/CD33^−^‐TKI), CD13/CD33 positive without TKI therapy group (CD13/CD33^+^‐non‐TKI), and CD13/CD33 positive with TKI treatment group (CD13/CD33^+^‐TKI).

The variables associated with CR1 included group (*p* < 0.001) and WBC (OR = 0.996, 95% CI: 0.994–0.999, *p* = 0.005). There were remarkable differences in CR1 between the CD13/CD33^−^‐TKI and CD13/CD33^−^‐non‐TKI subgroups (OR = 2.687, 95% CI: 1.234–5.851, *p* = 0.013) as well as between the CD13/CD33^+^‐TKI and CD13/CD33^+^‐non‐TKI subgroups (OR = 4.329, 95% CI: 2.092–8.929, *p* < 0.001).

For MRD detected by FCM, the variables associated were B‐ALL subtypes (*p* = 0.029) and age (OR = 0.987, 95% CI: 0.975–1.000, *p* = 0.043). Specifically, the pre‐B subtype showed a lower risk of MRD positivity than the common‐B (OR = 0.193, 95% CI: 0.055–0.671, *p* = 0.01) and pro‐B (OR = 0.232, 95% CI: 0.065–0.825, *p* = 0.024) subtypes.

For mortality, male sex (OR = 1.783, 95% CI: 1.073–2.963, *p* = 0.026), CD10 expression (OR = 0.509, 95% CI: 0.284–0.913, *p* = 0.023), and a high level of WBC (OR = 1.003, 95% CI: 1.000–1.005, *p* = 0.031) were independently associated with death.

The median RFS of patients in the CD13/CD33^−^‐TKI, CD13/CD33^−^‐non‐TKI, CD13/CD33^+^‐TKI, and CD13/CD33^+^‐non‐TKI subgroups was 7.5, 6, 9.5, and 6 months, respectively. In multivariate Cox regression analysis, group (*p* = 0.021) and WBC (HR = 1.003, 95% CI: 1.002–1.005, *p* < 0.001) were variables associated with RFS. Patients in the CD13/CD33^+^‐non‐TKI and CD13/CD33^−^‐non‐TKI subgroups suffered from a 1.754 times (95% CI: 1.130–2.754, *p* = 0.013) and 1.631 times (95% CI: 1.065–2.500, *p* = 0.025) higher risk of relapse than those in the CD13/CD33^+^‐TKI subgroup, respectively.

For OS, the median survival times in the CD13/CD33^−^‐TKI, CD13/CD33^−^‐non‐TKI, CD13/CD33^+^‐TKI, and CD13/CD33^+^‐non‐TKI subgroups were 9, 8, 12, and 9 months, respectively. In multivariate Cox regression analysis, three variables, including group, sex, and WBC count, were associated with the OS of B‐ALL patients (*p* = 0.048; *p* = 0.005; *p* < 0.001, respectively). The CD13/CD33^+^‐non‐TKI subgroup had an inferior OS than the CD13/CD33^+^‐TKI subgroup (HR = 2.676, 95% CI: 1.195–5.996, *p* = 0.017).

### 3.5 Cox regression model for survival prediction

**FIGURE 2:**
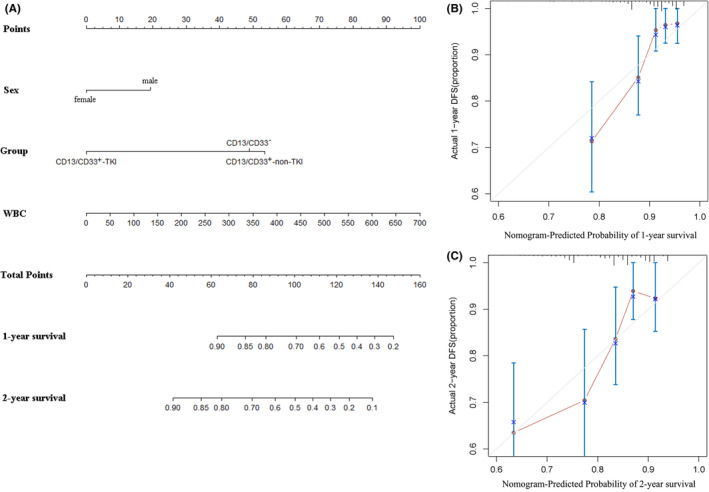
Cox regression model for survival prediction of patients with B‐cell acute lymphoblastic leukemia (B‐ALL). (A) Nomogram of the overall survival prediction model, (B) Calibration curves for the 1‐year survival of B‐ALL patients. The gray line represents the ideal performance, while the red line represents the actual performance of the model and (C) Calibration curves for the 2‐year survival of B‐ALL patients. The gray line represents the ideal performance, while the red line represents the actual performance of the model.

Next, we constructed a Cox regression model to predict the 1‐ and 2‐year survival for patients with B‐ALL. The CD13/CD33^−^‐TKI and CD13/CD33^−^‐non‐TKI subgroups were merged into CD13/CD33^−^ because they showed no significant difference in OS. Thus, the group was transformed from the original four‐categorical into a tripartite variable. Considering their associations with OS, sex, group based on CD13/CD33 expression and TKI experience as a four‐category variable and WBC count were finally incorporated into the model (Figure 2A). In internal validation, the C‐index of the nomogram based on these factors was 0.724, suggesting a moderate performance in survival prediction. Additionally, both calibration curves for predicting the probability of 1‐ and 2‐year survival showed consistency with the actual observations, with the latter being more accurate (Figure 2B,C).

## 4 DISCUSSION

In this real‐world study, we comprehensively analyzed the characteristics, outcomes, and prognostic factors of the largest adult B‐ALL cohort to date. Specifically, we explored the prognostic value of the MyAgs CD13/CD33 in adult B‐ALL patients. We found that CD13/CD33 was expressed in 53.7% of the total study subjects, which is higher than previous results on smaller adult cohorts.^21^, ^24^ The fluctuations in the MyAg^+^ ratio from various laboratories may be partially due to the varying standards for defining a positive event and the numbers of cases involved in each study. We also observed that CD13/CD33 expression was associated with a higher proportion of the common‐B subtype and lower levels of LDH and CD34 positivity. BCR::ABL1 was present in 45.6% of the 702 patients with molecular results available. This is higher than the earlier reported incidence of 20%–30% in Western populations.^25^ Although studies on Chinese adult B‐ALL patients are rare, it seems that the incidence of BCR::ABL1 rearrangement in this population is higher,^26^, ^27^ in line with our study. The development of more sensitive modalities to define the molecular and cytogenetic characteristics may also account for this difference.

Of note, CD13/CD33^+^ patients had a higher incidence of BCR::ABL1 positivity in our analysis, suggesting a concordant role with the previous study that measuring MyAg expression can predict the presence of BCR::ABL1 rearrangement.^24^ TKIs targeting the BCR::ABL1 oncogenic protein have been incorporated into front‐line treatment regimens for newly diagnosed Philadelphia (Ph)^+^ ALL.^28^ In univariate regression analysis, the CD13/CD33^+^ group had a higher incidence of MRD positivity than the CD13/CD33^−^ group. Furthermore, patients from the CD13/CD33^+^‐non‐TKI subgroup had a higher incidence of MRD positivity than those from the CD13/CD33^−^‐non‐TKI subgroup. These results support a novel role of CD13/CD33 expression in predicting the risk of MRD in patients without TKI experience. This is of clinical significance because MRD has been integrated into the algorithm of the treatment guidelines in evaluating the early treatment response and predicting outcome in multiple centers. Therefore, monitoring CD13/CD33 expression may facilitate more precise therapeutic decisions in B‐ALL management. While some laboratories have established standard antibody panels with^29^, ^30^ or without CD13/CD33^31^ for MRD measurements, our single‐center experience is to make CD13 and CD33 routine markers for both B‐ALL diagnosis and MRD detection.

The prognostic value of MyAg expression in adult ALL has been controversial. In our analysis, the CD13/CD33^+^ and CD13/CD33^−^ groups showed comparable incidences of CR1, CNS involvement, relapse and death, as well as time to CNS involvement, RFS and OS, suggesting that CD13/CD33 has no adverse effect on the clinical course, prognosis, and outcome of patients with B‐ALL. It seems that patients who underwent high‐dose chemotherapies^18^, ^19^, ^20^, ^21^ were prone to have similar outcomes regardless of MyAg expression, while earlier studies reported an inferior outcome of MyAg^+^ patients.^14^, ^15^, ^16^, ^17^ Vitale et al. reported that there were no differences between the MyAg^+^ and MyAg^−^ groups in terms of CR, DFS, and OS; however, the impact on MRD positivity was not measured, nor was the therapy incorporated with TKIs.^21^ Tong et al. reported that the expression of MyAg can predict the presence of BCR::ABL1 but not worsen outcome in a small number of Chinese adult ALL cohort receiving a VDLP‐based remission‐induction and consolidation therapy.^32^ Our results are consistent with these findings in that CD13/CD33 expression has no prognostic value for patient outcomes. However, the current study still outstands itself in that we incorporated the more modern TKI therapy into the variables to uncover the prognostic value of CD13/CD33 expression in a large real‐world adult B‐ALL cohort.

In multivariate analysis, the CD13/CD33^+^‐TKI subgroup showed a better prognosis, including a higher CR1, lower risk of mortality and relapse, and longer OS than the CD13/CD33^+^‐non‐TKI subgroup. This trend was not observed in comparisons between the CD13/CD33^−^‐TKI and CD13/CD33^−^‐non‐TKI subgroups. This may be due to the positive association of CD13/CD33 expression and BCR::ABL1, the target of TKIs. Therefore, our results strongly support that inclusion of TKIs into the therapy of Ph^+^ B‐ALL patients is beneficial.

While the use of standard protocols results in remission rates of 60%–92%, relapse remains a major obstacle for long‐term survival for adults with B‐ALL.^1^ Unfortunately, a prognostic model facilitating choices on treatment options in B‐ALL patients is under development. In this study, we constructed a prediction model incorporating variables independently associated with OS, including sex, age, and group featuring CD13/CD33 expression and TKI experience, through Cox regression analysis. The application of this model displaying moderate performance should be accompanied with caution. The prognosis of adult B‐ALL patients can be simultaneously affected by the BM microenvironment,^33^, ^34^ plasma/serum biomarkers,^35^ associations with molecular events and cytogenetics,^36^ and varied regimens.^37^ The variables were limited to three in the present study due to its retrospective nature. Future studies on models with more variables independently associated with prognosis via more modern methods other than traditional statistical methods may be required to reach a better predicting performance. Above all, external validation of the prediction model on a different cohort should be performed.

To the best of our knowledge, the current study is the largest real‐world single‐center presentation of adult B‐ALL patients to date. However, there are limitations. First, this study lies in its retrospective nature and its heterogeneity in baseline risk and treatment factors as a real‐world one, which may have led to potential bias. Furthermore, there are inherent drawbacks as a single‐center study. These results need to be further validated in larger prospective studies to clarify the mechanisms.

## 5 CONCLUSIONS

In summary, our large real‐world study demonstrated that cross‐lineage expression of the myeloid antigen CD13/CD33 in adult B‐ALL can predict the risk of MRD positivity and the presence of the BCR::ABL1/Ph chromosome but has no adverse effect on patient outcomes. Targeted TKI therapy can improve the prognosis of Ph^+^ B‐ALL patients. Therefore, we propose that CD13/CD33 should be set as routine markers in the standard antibody panels in the diagnosis and MRD measurement of B‐ALL. More variables can be included in our prediction model originally based on WBC, group,and sex to reach a better performance for predicting the OS of B‐ALL patients. Further analysis of the gene expression patterns for adult B‐ALL patients with and without CD13/CD33 may provide new potential pathological mechanisms and therapeutic targets.

## AUTHOR CONTRIBUTIONS

**Hongyan Liao:** Conceptualization (equal); funding acquisition (equal); validation (equal); writing – original draft (lead); writing – review and editing (lead). **Hongli Lai:** Formal analysis (equal); methodology (lead); visualization (lead); writing – original draft (supporting); writing – review and editing (supporting). **Jiao Chen:** Investigation (equal); resources (equal); writing – review and editing (supporting). **Xiao Shuai:** Data curation (supporting); methodology (supporting); validation (lead); writing – review and editing (supporting). **Xin Zhang:** Investigation (equal); resources (equal); writing – review and editing (supporting). **Ying Yang:** Investigation (supporting); resources (supporting); writing – review and editing (supporting). **Mengyuan Lyu:** Formal analysis (supporting); validation (supporting); writing – review and editing (supporting). **Qin Zheng:** Conceptualization (equal); funding acquisition (equal); project administration (equal); resources (equal); supervision (lead); writing – original draft (supporting); writing – review and editing (supporting).

## CONFLICT OF INTEREST STATEMENT

The authors have no conflicts of interest to declare.

## Supporting information


**Table S1.** Basic characteristics of adult B‐ALL patients.

**Table S2.** Antibody panels for flow cytometry analysis

**Table S3.** Univariate regression analysis of CD13/CD33 and BCR::ABL1 on the prognosis of the B‐ALL patient group.

## Data Availability

Research data are stored in an institutional repository. Secondary use of study data will be considered upon a reasonable request and approval of IRB.
