# Supplementary material for: Prognostic value of cross‐lineage expression of the myeloid‐associated antigens CD13 and CD33 in adult B‐lymphoblastic leukemia: A large real‐world study of 1005 patients
Source: Cancer Med. 2023 Mar 23;12(8):9615–26. doi: 10.1002/cam4.5739 (PMC10166937; doi:10.1002/cam4.5739)
Supplement: Supplementary file 1 — Table S1. Basic characteristics of adult B‐ALL patients. [file CAM4-12-9615-s002.docx]

**Supplementary Table 1. Basic characteristics of adult B-ALL patients.**

| **Characteristics** | **All included patients (n=1005)** | | | |
| --- | --- | --- | --- | --- |
|  | **Overall** | **CD13/CD33^+^**  **(n=540)** | **CD13/CD33^-^**  **(n=465)** | **P value** |
| **General characteristics** | | | | |
| Sex |  |  |  | 0.215 |
| Male, n (%) | 501 (49.9) | 279 (51.7) | 222 (47.7) |  |
| Female, n (%) | 504 (50.1) | 261 (48.3) | 243 (52.3) |  |
| Age, median (range), years | 35.00 (22.00-46.00) | 35.00 (23.75-47.00) | 34.00 (21.00-45.00) | 0.532 |
| B-ALL subtype |  |  |  | **0.001** |
| pro-B, n (%) | 347 (34.5) | 184 (34.1) | 163 (35.1) |  |
| common-B, n (%) | 588 (58.5) | 333 (61.7) | 255 (54.8) |  |
| pre-B, n (%) | 70 (7.0) | 23 (4.3) | 47 (10.1) |  |
| **Laboratory indices** | | | | |
| Hb, g/L | 77.00 (62.00-100.75) | 76.00 (61.00-99.00) | 79.00 (63.00-104.50) | 0.235 |
| PLT, ×10^9^/L | 38.00 (19.00-81.75) | 38.00 (19.00-85.00) | 37.00 (19.00-75.25) | 0.386 |
| WBC, ×10^9^/L | 11.85 (3.35-54.14) | 13.08 (4.01-54.21) | 10.69 (3.03-55.14) | 0.082 |
| PB-BLA, ×10^9^/L | 4.57 (0.06-44.01) | 5.57 (0.143-41.96) | 3.07 (0.00-44.46) | 0.107 |
| BM-BLA, % | 83.50 (70.50-91.00) | 83.00 (72.00-91.00) | 84.000 (68.38-91.50) | 0.950 |
| LDH, IU/L | 532.50 (292.00-1058.50) | 506.00 (275.50-907.75) | 555.00 (298.75-1383.25) | **0.030** |
| CD34^+^, n (%) | 492 (89.6) | 269 (94.4) | 223 (84.5) | **<0.001** |
| CD10^+^, n (%) | 818 (81.4) | 449 (83.1) | 369 (79.4) | 0.123 |
| CD13^+^, n (%) | 492 (49.0) | / | / | **/** |
| CD33^+^, n (%) | 227 (22.6) | / | / | **/** |
| BCR::ABL1^+^, n (%) | 320 (45.6) | 206 (54.5) | 114 (35.2) | **<0.001** |
| P190/P210 |  |  |  | 0.616 |
| Both -, n (%) | 23 (28.8) | 15 (29.4) | 8 (27.6) |  |
| P190, n (%) | 31 (38.8) | 21 (41.2) | 10 (34.5) |  |
| P210, n (%) | 25 (31.2) | 15 (29.4) | 10 (34.5) |  |
| Both +, n (%) | 1 (1.2) | 0 (0) | 1 (3.4) |  |
| Other molecular cytogenetics |  |  |  | **0.045** |
| MLL-AF4, n (%) | 26 (33.3) | 6 (21.4) | 20 (40) |  |
| TEL-AML1, n (%) | 6 (7.7) | 5 (17.9) | 1 (2) |  |
| E2A-PBX1, n (%) | 31 (39.8) | 10 (35.7) | 21 (42) |  |
| HOX11, n (%) | 5 (6.4) | 1 (3.6) | 4 (8) |  |
| AML-related, n (%) | 5 (6.4) | 3 (10.7) | 2 (4) |  |
| others, n (%) | 5 (6.4) | 3 (10.7) | 2 (4) |  |

Abbreviations: B-ALL, B cell acute lymphoblastic leukemia; WBC, white blood cell; PLT, platelet; Hb, hemoglobin; PB-BLA, blast cells in peripheral blood; BM-BLA, blast cells in bone marrow; LDH, lactate dehydrogenase; AML-related, acute myeloid leukemia-related.

Categorical variables are presented as numbers (percentages). Continuous variables without normal distributions were expressed as the median with the 25th and 75th percentiles.
