# Supplementary material for: Prognostic value of cross‐lineage expression of the myeloid‐associated antigens CD13 and CD33 in adult B‐lymphoblastic leukemia: A large real‐world study of 1005 patients
Source: Cancer Med. 2023 Mar 23;12(8):9615–26. doi: 10.1002/cam4.5739 (PMC10166937; doi:10.1002/cam4.5739)
Supplement: Supplementary file 2 — Table S2. Antibody panels for flow cytometry analysis [file CAM4-12-9615-s001.docx]

**Supplementary Table 2. Antibody Panels for Flow Cytometry Analysis**

| **Marker** | **Fluorochrome** |
| --- | --- |
| **5-tube-4-color** |  |
| Tube 1 |  |
| CD5 | APC |
| CD19 | PE |
| CD38 | FITC |
| CD45 | PerCP |
| Tube 2 |  |
| CD10 | FITC |
| CD20 | APC |
| CD22 | PE |
| CD45 | PerCP |
| Tube 3 |  |
| CD34 | FITC |
| CD45 | PerCP |
| CD117 | PE |
| HLA-DR | APC |
| Tube 4 |  |
| CD13 | PE |
| CD33 | APC |
| CD45 | PerCP |
| Tube 5 |  |
| cCD3 | FITC |
| cMPO | PE |
| cCD79a | PerCP |
| CD45 | APC |
| **1-tube-8-color** |  |
| CD10 | FITC |
| CD13 | PE |
| CD33 | PE |
| CD19 | BV510 |
| CD20 | APC |
| CD34 | PE-Cy7 |
| CD38 | BV421 |
| CD45 | PerCP |
| HLA-DR | APC-Cy7 |
