# Supplementary material for: Prognostic value of cross‐lineage expression of the myeloid‐associated antigens CD13 and CD33 in adult B‐lymphoblastic leukemia: A large real‐world study of 1005 patients
Source: Cancer Med. 2023 Mar 23;12(8):9615–26. doi: 10.1002/cam4.5739 (PMC10166937; doi:10.1002/cam4.5739)
Supplement: Supplementary file 3 — Table S3. Univariate regression analysis of CD13/CD33 and BCR::ABL1 on the prognosis of the B‐ALL patient group. [file CAM4-12-9615-s003.docx]

**Supplementary Table 3. Univariate regression analysis of CD13/CD33 and BCR::ABL1 on the prognosis of the B-ALL patient group.**

|  | **Outcome measures** | **Variable** | **OR/HR (95% CI)** | **P value** |
| --- | --- | --- | --- | --- |
| **Univariate Logistic regression** | **CR1** | Group^a^ | / | **0.145** |
|  |  | CD13/CD33^-^-BCR::ABL1^+^ vs CD13/CD33^-^-BCR::ABL1^-^ | 0.820 (0.385-1.746) | 0.607 |
|  |  | CD13/CD33^+^-BCR::ABL1^-^ vs CD13/CD33^-^-BCR::ABL1^-^ | 0.594 (0.305-1.156) | 0.125 |
|  |  | CD13/CD33^+^-BCR::ABL1^+^ vs CD13/CD33^-^-BCR::ABL1^-^ | 1.305 (0.648-2.628) | 0.457 |
|  |  | CD13/CD33^-^-BCR::ABL1^+^ vs CD13/CD33^+^-BCR::ABL1^+^ | 0.629 (0.290-1.365) | 0.241 |
|  |  | CD13/CD33^+^-BCR::ABL1^-^ vs CD13/CD33^+^-BCR::ABL1^+^ | **0.455 (0.229-0.906)** | **0.025** |
|  |  | CD13/CD33^+^-BCR::ABL1^-^ vs CD13/CD33^-^-BCR::ABL1^+^ | 0.724 (0.344-1.524) | 0.395 |
|  | **MRD** | Group | / | 0.427 |
|  |  | CD13/CD33^-^-BCR::ABL1^+^ vs CD13/CD33^-^-BCR::ABL1^-^ | 1.208 (0.663-2.204) | 0.537 |
|  |  | CD13/CD33^+^-BCR::ABL1^-^ vs CD13/CD33^-^-BCR::ABL1^-^ | 1.534 (0.876-2.685) | 0.134 |
|  |  | CD13/CD33^+^-BCR::ABL1^+^ vs CD13/CD33^-^-BCR::ABL1^-^ | 1.420 (0.848-2.377) | 0.182 |
|  |  | CD13/CD33^-^-BCR::ABL1^+^ vs CD13/CD33^+^-BCR::ABL1^+^ | 0.851 (0.473-1.529) | 0.589 |
|  |  | CD13/CD33^+^-BCR::ABL1^-^ vs CD13/CD33^+^-BCR::ABL1^+^ | 1.080 (0.627-1.861) | 0.781 |
|  |  | CD13/CD33^+^-BCR::ABL1^-^ vs CD13/CD33^-^-BCR::ABL1^+^ | 1.270 (0.679-2.374) | 0.455 |
|  | **Relapse rate** | Group | / | 0.076 |
|  |  | CD13/CD33^-^-BCR::ABL1^+^ vs CD13/CD33^-^-BCR::ABL1^-^ | **0.533 (0.285-0.995)** | **0.048** |
|  |  | CD13/CD33^+^-BCR::ABL1^-^ vs CD13/CD33^-^-BCR::ABL1^-^ | 0.982 (0.532-1.812) | 0.954 |
|  |  | CD13/CD33^+^-BCR::ABL1^+^ vs CD13/CD33^-^-BCR::ABL1^-^ | 0.586 (0.339-1.014) | 0.056 |
|  |  | CD13/CD33^-^-BCR::ABL1^+^ vs CD13/CD33^+^-BCR::ABL1^+^ | 0.910 (0.495-1.674) | 0.761 |
|  |  | CD13/CD33^+^-BCR::ABL1^-^ vs CD13/CD33^+^-BCR::ABL1^+^ | 1.676 (0.922-3.046) | 0.090 |
|  |  | CD13/CD33^+^-BCR::ABL1^-^ vs CD13/CD33^-^-BCR::ABL1^+^ | 1.842 (0.944-3.594) | 0.073 |
|  | **Mortality** | Group | / | 0.284 |
|  |  | CD13/CD33^-^-BCR::ABL1^+^ vs CD13/CD33^-^-BCR::ABL1^-^ | 0.641 (0.291-1.411) | 0.269 |
|  |  | CD13/CD33^+^-BCR::ABL1^-^ vs CD13/CD33^-^-BCR::ABL1^-^ | 0.726 (0.367-1.436) | 0.357 |
|  |  | CD13/CD33^+^-BCR::ABL1^+^ vs CD13/CD33^-^-BCR::ABL1^-^ | 0.509 (0.252-1.027) | 0.059 |
|  |  | CD13/CD33^-^-BCR::ABL1^+^ vs CD13/CD33^+^-BCR::ABL1^+^ | 1.259 (0.535-2.965) | 0.598 |
|  |  | CD13/CD33^+^-BCR::ABL1^-^ vs CD13/CD33^+^-BCR::ABL1^+^ | 1.426 (0.668-3.046) | 0.359 |
|  |  | CD13/CD33^+^-BCR::ABL1^-^ vs CD13/CD33^-^-BCR::ABL1^+^ | 1.133 (0.489-2.625) | 0.771 |
| **Univariate Cox regression** | **RFS** | Group | / | 0.120 |
|  |  | CD13/CD33^-^-BCR::ABL1^+^ vs CD13/CD33^-^-BCR::ABL1^-^ | **0.599 (0.364-0.984)** | **0.043** |
|  |  | CD13/CD33^+^-BCR::ABL1^-^ vs CD13/CD33^-^-BCR::ABL1^-^ | 1.001 (0.665-1.507) | 0.997 |
|  |  | CD13/CD33^+^-BCR::ABL1^+^ vs CD13/CD33^-^-BCR::ABL1^-^ | 0.754 (0.510-1.114) | 0.156 |
|  |  | CD13/CD33^-^-BCR::ABL1^+^ vs CD13/CD33^+^-BCR::ABL1^+^ | 0.795 (0.478-1.321) | 0.375 |
|  |  | CD13/CD33^+^-BCR::ABL1^-^ vs CD13/CD33^+^-BCR::ABL1^+^ | 1.328 (0.870-2.026) | 0.188 |
|  |  | CD13/CD33^+^-BCR::ABL1^-^ vs CD13/CD33^-^-BCR::ABL1^+^ | 1.671 (0.993-2.813) | 0.053 |
|  | **OS** | Group | / | 0.199 |
|  |  | CD13/CD33^-^-BCR::ABL1^+^ vs CD13/CD33^-^-BCR::ABL1^-^ | 0.595 (0.276-1.282) | 0.185 |
|  |  | CD13/CD33^+^-BCR::ABL1^-^ vs CD13/CD33^-^-BCR::ABL1^-^ | 0.709 (0.372-1.353) | 0.298 |
|  |  | CD13/CD33^+^-BCR::ABL1^+^ vs CD13/CD33^-^-BCR::ABL1^-^ | **0.498 (0.254-0.979)** | **0.043** |
|  |  | CD13/CD33^-^-BCR::ABL1^+^ vs CD13/CD33^+^-BCR::ABL1^+^ | 1.194 (0.510-2.798) | 0.682 |
|  |  | CD13/CD33^+^-BCR::ABL1^-^ vs CD13/CD33^+^-BCR::ABL1^+^ | 1.424 (0.677-2.994) | 0.351 |
|  |  | CD13/CD33^+^-BCR::ABL1^-^ vs CD13/CD33^-^-BCR::ABL1^+^ | 1.192 (0.521-2.726) | 0.677 |

Abbreviations: CR1, complete remission after first induction; MRD, minimal residual disease; OS, overall survival; OR, odds ratio; HR, hazard ratio; CI, confidence interval.

a According to CD13/CD33 expression and BCR::ABL1 expression, patients were categorized into four groups: CD13/CD33 and BCR::ABL1 both negative (CD13/CD33^-^- BCR::ABL1^-^), CD13/CD33 negative and BCR::ABL1 positive (CD13/CD33^-^- BCR::ABL1^+^), CD13/CD33 positive and BCR::ABL1 negative (CD13/CD33^+^- BCR::ABL1^-^), and CD13/CD33 and BCR::ABL1 both positive (CD13/CD33^+^- BCR::ABL1^+^).
